# Combined treatment with CDK4/6, CDK2, and CXCR1/2 inhibitors effectively halts the growth of BRAF wild-type melanoma tumors

**DOI:** 10.3389/fonc.2025.1609735

**Published:** 2025-08-19

**Authors:** Jinming Yang, Weifeng Luo, Patricia Ward, Sheau-Chiann Chen, John Zebala, Dean Maeda, Chi Yan, Ann Richmond

**Affiliations:** ^1^ Tennessee Valley Healthcare System (TVHS) Department of Veterans Affairs, Nashville, TN, United States; ^2^ Department of Pharmacology, Vanderbilt University, Nashville, TN, United States; ^3^ Department of Biostatistics, Vanderbilt University Medical Center, Nashville, TN, United States; ^4^ Syntrix Biosystems, Auburn, WA, United States; ^5^ Department of Immunology, Max Rady College of Medicine and Rady Faculty of Health Sciences, University of Manitoba, Winnipeg, MB, Canada; ^6^ Paul Albrechtsen Research Institute, CancerCare Manitoba, Winnipeg, MB, Canada

**Keywords:** CDK inhibitors, CXCR2 antagonist, tumor immune microenvironment, tumor growth, melanoma

## Abstract

**Introduction:**

Inhibitors of cyclin-dependent kinase 4 and 6 (CDK4/6) are approved for the treatment of locally advanced or metastatic breast cancer, but not for melanoma.

**Methods:**

In this study, we evaluated the effectiveness of the CDK4/6 inhibitor, palbociclib, the CDK2 inhibitor, PF-07104091, the dual CXCR1 and CXCR2 (CXCR1/2) antagonist, SX-682, and the combination of these inhibitors for effective treatment of melanoma in preclinical models.

**Results:**

Both palbociclib and SX-682 inhibited the growth of BRAF^WT^/NRAS^WT^ B16-F10 and NRAS^mut^ 1014 melanoma tumors and in both models, SX-682 created a more anti-tumor immune microenvironment. The combination effect was additive in the B16F10 model, but not in the 1014 model. In the B16F10 model, the addition of the CDK2 inhibitor, PF-07104091, overcame B16F10 acquired resistance to CDK4/6 inhibitors by suppressing the induction of cyclin D1 and E1 expression by palbociclib. In the less responsive 1014 cells, cyclin D1 was reduced, but cyclin E1 was induced in response to PF-07104091. However, in both models, combined treatment with palbociclib and PF-07104091 markedly suppressed cyclin A2, cyclin D1, cyclin E1 and pRB-S807/S811. Combining CDK4/6 and CDK2 inhibitors with the CXCR1/2 antagonist, SX-682, halted B16F10 tumor growth by blocking tumor cell proliferation and increasing the anti-tumor immune response in the tumor microenvironment.

**Conclusions:**

The combination of all three inhibitors resulted in a tumor microenvironment characterized by increased IFNγ-producing CD4+ T cells, decreased CD4+FOXP3+ T regulatory cells (Tregs), and decreased IL-10-producing CD4+ T cells. This combination also decreased the percentage of CD8+ T cells that expressed PD-1 or TIM-3 and increased the ratio of MHCII+F4/80+ M1-like macrophages to CD206+F4/80+ M2-like macrophages. These data suggest that inhibiting CDK4/6 and CDK2, combined with antagonism of CXCR1/2, may be an effective treatment for BRAF wild-type melanoma tumors and NRAS mutant melanoma tumors that express Rb and are resistant to immune checkpoint inhibitors.

## 1 Introduction

Metastatic melanoma has the highest death rate in relation to incidence of any skin cancer (1). Recent advances in treatment have led to significant improvements in the outcomes for patients with melanoma. Immune checkpoint inhibitors (ICI) are the standard of care, with combined nivolumab and ipilimumab treatment yielding a median overall survival (OS) of 71.9 months in untreated advanced melanoma (2). Recently, a phase 2 trial involving 333 patients revealed that neo-adjuvant treatment of resectable stage III or stage IV melanoma with pembrolizumab significantly improved event-free survival (72%) compared to adjuvant-only treatment (49%) after 2 years (3). Despite the clinical success of ICI in advanced melanoma, outcomes remain sobering, with more than half of patients eventually progressing, after which median OS is only about 6 months (4, 5). The need for improved treatments thus remains high. It has been demonstrated that ICI is less effective for those melanoma tumors with an immunosuppressive tumor microenvironment (TME) (6). For metastatic melanoma patients who progress on ICI therapy, BRAF/MEK inhibitors offer an additional follow-up therapy option for patients with BRAF-mutant (BRAF^V600E^) advanced melanoma. Adjuvant treatment of stage III resected melanoma with BRAF/MEK inhibitors provided a 71% OS at 8 years and a median progression-free survival (PFS) of 93.1 months. However, treatment with the BRAF inhibitor encorafenib plus the MEK inhibitor binimetinib provided only a seven-year 21.2% PFS and 27.4% OS in 192 patients with unresectable or metastatic BRAF-mutant melanoma who were treatment-naïve or had progressed on first-line immunotherapy (7). Another study of 589 stage III BRAF-mutant melanoma patients revealed that treatment with anti-PD1 monotherapy resulted in a similar OS compared to treatment with dabrafenib plus trametinib, and the median relapse-free survival was 51 months in the dabrafenib + trametinib treatment group compared to 44.8 months in the anti-PD-1 monotherapy group (8). However, based upon the results of the DREAMseq study, BRAF-mutant melanoma patients should first be treated with ICI, and if they progress, BRAF/MEK inhibitors can be highly successful (9).

In contrast, there are limited treatment choices for patients with NRAS-mutant or BRAF^WT^/NRAS^WT^ melanoma who progress after ICI therapy (i.e., *de novo* or acquired resistance). Based on the observation that the CDK4/6 pathway is frequently altered in melanoma, CDK4/6 inhibitors have emerged as potential agents for treating patients with NRAS-mutant and BRAF-wild-type tumors that have not lost the tumor suppressor retinoblastoma protein (pRb) (10, 11). Indeed, two ongoing clinical trials are evaluating the efficacy of CDK4/6 inhibitors, particularly when combined with a MEK inhibitor as part of a multidrug regime (NCT04720768 and NCT02645149) (12). CDK4/6 inhibitors have been reported to augment the anti-tumor memory T cell pool and improve the response to subsequent anti-PD-1 therapy, expanding the T effector population (13). However, sequential, rather than simultaneous, treatment of CDK4/6 inhibitors with anti-PD-1 or BRAF/MEK therapy is advised to optimize the positive and mitigate the adverse immunomodulatory effects of each treatment on the TME (14). Resistance mechanisms to CDK4/6 inhibition have been identified, which involve the induction of Cyclin D1, which sequesters p21 and p27, thereby leaving CDK2 uninhibited. Additionally, resistance to CDK4/6 inhibitors in breast cancer is acquired through CDK2-mediated phosphorylation of c-MYC, which enables cells to escape senescence. Thus, the synergistic antiproliferative effect of the combined inhibition of CDK2 and CDK4/6 in breast cancer can overcome acquired resistance to CDK4/6 inhibitors by enhancing senescence (15).

The gene encoding the cyclin-dependent kinase inhibitor 2A (CDKN2A) is frequently lost or mutated in 40-70% of sporadic melanoma tumors, and 20-40% of familial melanomas (10). It has been shown that melanoma tumors with loss of CDKN2A are often highly sensitive to CDK4/6 inhibition (16). We have previously demonstrated that combined treatment with a CDK4/6 inhibitor and an MDM2 inhibitor suppresses the growth of melanoma patient-derived xenografts and that knockdown of CDK2 overcomes resistance to CDK4/6 inhibition (17).

Melanoma tumors often exhibit an immune suppressive TME and secrete various cytokines and chemokines that are key signals involved in the recruitment of MDSCs. MDSCs express the CXC chemokine receptor 1 and 2 (CXCR1/2), and their ligands are produced by melanoma cells (18). Thus, CXCR1/2 not only regulates neutrophil trafficking from the bone marrow to peripheral circulation or inflammation sites (19) but also plays a role in tumor progression by facilitating the migration of tumor-associated myeloid cells into the TME (20). Therefore, targeting CXCR1/2 should alter the accumulation of tumor-associated myeloid cells and MDSCs in the TME, favoring a less immune-suppressive TME (21, 22). We have previously demonstrated that the treatment of mice bearing genetically derived inducible melanoma tumors (BRAF/PTEN or NRAS/INK4a) with the CXCR1/2 antagonist (SX-682) inhibited tumor growth and increased activated CD8+ T cells, partly by reducing the intratumoral MDSCs (6). There are ongoing clinical trials combining SX-682 with anti-PD-1 to treat advanced metastatic melanoma (23). These compelling studies from solid tumors in either mice or humans prompted us to investigate the therapeutic potential of combining CDK inhibitors and SX-682 for treating NRAS^mut^ and BRAF^WT^ melanoma. Here, we initiate a preclinical study using murine BRAF^WT^ murine B16-F10 melanoma and NRAS^mut^ 1014 melanoma cells to examine the effectiveness of combining the CXCR1/2 antagonist, SX-682, with the CDK4/6 inhibitor, palbociclib. We also evaluate the addition of the CDK2 inhibitor, tagtociclib (PF-07104091) (24), to this treatment regimen and assess whether this triple- drug combination results in greater inhibition of tumor growth in the B16F10 mouse model of melanoma.

## 2 Materials and methods

### 2.1 Mouse melanoma tumor model

All mouse experiments were performed under a protocol approved by the Vanderbilt IACUC committee (#M2000008), and guidelines were strictly adhered to. C57/Bl6 female mice of 8-10 weeks old were purchased from Charles River (Wilmington, MA, RRID: IMSR_CRL:027). B16-F10 melanoma cells (ATCC, RRID : CVCL_0159) that carry amplifications of *Braf* and *Met* oncogenes and missense mutations of the tumor suppressor *Pten*, and loss of CDKNA (25) or 1014 NRAS^Q61K/^PTEN^WT^/CDKN2A ^WT^ expressing melanoma cells (the kind gift of Lionel Larue, Institut Curie, Centre Universitaire (26), were examined for mycoplasma contamination monthly and any contaminated cultures were discarded. Authentication of genotypes for these cell lines was determined by DNA sequence analysis. Tumor cells were implanted subcutaneously on both sides of the intrascapular region of the mouse (3 x 10^5^ for B16F10 and 3 x10^5^ for 1014 cells). When the tumor size reached approximately 125mm^3^, mice were randomly assigned to two groups: half the tumor-bearing mice were fed regular chow, and the other half were fed chow containing SX-682 (1,428 mg/kg of chow), as previously described, but at a dose was twice that previously used (6). The toxicity and plasma levels of SX-682 administered PO *ad libitum* in chow have been previously described (6, 27, 28). We have previously compared the effects of SX-682 treatment to the targeted knock out of CXCR2 on myeloid cells or melanocytic cells, where treatment with SX-682 had similar effects on tumor growth as targeted knock out of CXCR2, indicating good drug delivery (6, 27). Half the mice in each chow-fed treatment group were randomly assigned to receive oral gavage containing either vehicle alone or palbociclib (100 mg/kg bodyweight) daily for 5 days per week. In some experiments, mice received both palbociclib (100 mg/kg) and the CDK2 inhibitor PF-07104091 (50 mg/kg) in two separate gavages with or without SX-682-containing chow. When two different vehicle controls were used, two separate vehicle gavages were administered. The body weight and tumor size of mice were measured twice a week. Tumor volume was determined as 0.5 x length x width x width. Power analysis indicated that an n of 5 mice per group provided sufficient power to detect differences with a p-value ≤ 0.05 approximately 80% of the time. Only female mice were used due to the issue of male mice fighting if not bred as siblings.

### 2.2 Reagents

Palbociclib-HCL (PD-0332991)(Cat# S1116, Selleckchem, 99.82% purity) was dissolved in water (50°C, 100 mg/10 ml). The CDK2 inhibitor, tagtociclib (PF-07104091) (Cat#CT-PF0710, Chemietek, >99% pure) was dissolved in DMSO (81 mg/ml) for *in vitro* experiments. For *in vivo* experiments, PF-07104091 was dissolved in 5% DMSO, 40% PEG 300, 5% Tween 80, and 50% water. In addition, chow containing SX-682 (1,428 mg/kg of chow, Syntrix Pharmaceuticals) or chow containing vehicle control was used as previously described (27). Monoclonal antibodies (mAb) to phospho-Rb (Ser807/811) (D20B12, XP^®^ rabbit mAb Cat#8516, RRID: AB_11178658), Cyclin D1 (E3P5S, XP^®^ rabbit mAb Cat#55506, RRID: AB_2827374), Cyclin A2 (E9Q5G, rabbit mAb Cat#81754), and Cyclin E1 (D7T3U, rabbit mAb #20808) were purchased from Cell Signaling Technology.

### 2.3 Flow cytometry analysis and antibodies

For flow cytometry, tissues were minced on a programmable gentleMACS dissociator (Miltenyi Biotec, USA) and digested with an enzyme solution of collagenase 1 (1,500 CDU, CAT#234153, Calbiochem), dispase II (1 mg/mL, CAT#13689500, Roche), and DNase I (0.1 mg/mL, CAT#260913, Calbiochem). Staining and analysis protocols were according to our previously published methodology (6). Cells were incubated with Ghost Dye TM Violet 510 (Tonbo Biosciences), an amine-reactive viability dye used to discriminate live/dead cells and then washed with fluorescence-activated cell sorting (FACS) buffer (PBS containing 2% v/v FBS). After blocking Fc receptors with anti-mouse CD16/CD32 (BioLegend) in FACS buffer for 15 minutes, cells were incubated with fluorescence-conjugated mAbs specific to mouse immune cell surface or intracellular markers (BD Cytofix/Cytoperm Plus Kit#554715), indicated below for an additional 30 minutes on ice. Cells were washed twice in FACS buffer (23), data were acquired with a FACSCanto II flow cytometer (Becton Dickinson), and data (FCS files) were analyzed using FlowJo software (Version 10.1, RRID: SCR_008520). For cell-surface markers, the following monoclonal antibodies (mAbs) from eBioscience were used: CD11b-FITC (clone M1/70), CD3-FITC (clone 17A2), CD3-PECy5 (clone 17A2), CD103-Brilliant Violet 421 (clone 2E7), CD4-Pacific Blue (clone RM4–4), CD4-APC/Cy7 (clone RM4–4), CD8-PECy7 (clone 53–5.8), CD11c-APC (clone N418), CD19-PECy7 (clone N418), B220-APC (clone RA3–6B2), CD45-PerCp/Cy5.5 (clone 30-F11), CD45-APC/Cy7 (clone 30-F11), CD44-APC/Cy7 (clone IM7), NK1.1-APC/Cy7 (clone PK136), F4/80-Pacific Blue (clone BM8), CD69-APC (clone H1.2F3), CD107b-Alexa Fluor 647 (clone M3/84), CD62L-Alexa Fluor 647 (clone MEL-14), Ly6G-APC (clone 1A8), Ly6C-Alexa Fluor 647 (clone HK1.4), CD25-PerCp/Cy5.5 (clone 3C7), CD44-APC (clone IM7), MHC II-Alexa Fluor 647 (clone AF6–120.1), CD206-PE (clone C068C2), PD-1-APC/Cy7 (clone 29F.1A12), PD-L1-APC (clone 10F.9G2). For intracellular markers, monoclonal antibodies (mAbs) from BioLegend were used as follows: Foxp3-Alexa Fluor 647 (clone 150D), and Ki67-Pacific Blue (clone 16A8). We also used the following antibodies: IFNγ-Alexa Fluor 700 (clone XMG1.2, from BD Bioscience); Gost Dye violet 450, Gost Dye violet 510, and Gost Dye violet 780 (from Tonbo Biosciences).

### 2.4 Western blot analysis

After culturing 1014 or B16F10 melanoma cells in DMEM/F12 (1:1) with 10% FBS to 75% confluence, cells were treated with 20 μM Palbociclib and 20 μM PF-07104091 for 24 hours. Media was removed, and the cells were rinsed with cold PBS. The whole-cell lysates were prepared using RIPA buffer (Fisher Scientific, Cat. # PI89900) supplemented with a protease inhibitor cocktail (Roche, Cat. # 04693124001) and a phosphatase inhibitor cocktail (Roche, Cat. # 4906845001). The protein concentration was measured using Pierce™ BCA Protein Assay Kits (Thermo Scientific, Cat. # 23225). 40 μg of protein was separated on 4–20% Precast Midi Protein Gel (BioRad, Cat. # 5671094) and transferred to nitrocellulose membrane using Trans-Blot Turbo RTA Transfer Kit (Nitrocellulose, BioRad, Cat. # 1704272). Blots were blocked with Intercept Blocking Buffer (TBS) (LI-COR, Cat. # 927 60001) for 1 hour. Primary antibodies were diluted with Intercept Antibody Diluent (TBS) (LI-COR, Cat. # 927 65001). Blots were incubated with primary antibodies overnight at 4°C. These antibodies were purchased from Cell Signaling Technology: Cyclin A2 (Cat. # 81754), Cyclin D1 (Cat. # 55506), Cyclin E1 (Cat. # 20808), phospho-Rb S807/S811 (Cat. # 8516), and Caspase-3 (Cat. # 9662). Beta actin antibody was from Invitrogen (Cat. # MA5-15739). These secondary antibodies were used: IRDye^®^ 800CW Goat anti-Rabbit IgG (H + L) (LI-COR, Cat. # 926-32211), IRDye^®^ 680RD Goat anti-Mouse IgG (LI-COR, Cat. # 926-68070). Protein bands were visualized using the Odyssey CLx Imager (LI-COR). To detect cleaved caspase-3, the ECL Western blotting method was used. Blot was blocked with 5% non-fat milk in TBS buffer with 0.1% Tween-20 for 1 hour, then incubated with anti-cleaved caspase-3 (Cell Signaling Technology, Cat. # 9661) overnight at 4°C. After washing, the blot was incubated with the secondary antibody anti rabbit IgG-HRP (Cell Signaling Technology, Cat. # 7074P2) for 1 hour. After washing five times, the blot was incubated with SuperSignal™ West Pico PLUS Chemiluminescent Substrate for 5 minutes, and signals were captured using HyBlot CL^®^ Autoradiography Film (Thomas Scientific). All western blot bands were quantified with Image Studio software, and data were statistically analyzed using One-way ANOVA and Tukey’s test.

### 2.5 Cell cycle analysis

Cell cycle analysis by flow cytometry was based on measurement of DNA content by staining with propidium iodide (PI). Melanoma cells (B16-F10 and 1014) were plated in 6-well plates in DMEM/F-12 medium (Gibco, Cat. # 11330-032) + 10% FBS (Atlas Biologicals, Cat. # F-0500-A). When cell confluence reached approximately 80%, cells were treated with either 20 μM palbociclib alone, or 20 μM PF-07104091 alone, or 20 μM palbociclib plus 20 μM PF-07104091, or with vehicle control. After 24 hours of treatment, cells were trypsinized, and the cells in the media were also collected. After washing once with PBS, cells were fixed with 70% ethanol for 48 hours at -20°C. Cells were washed once, and the cells were counted for each sample. Cells were stained for 24 hours at 4°C at 1 x10^6^/ml with PI stain solution [PBS buffer with 0.1% Triton-X100, 200 μg/ml RNase (Qiagen, Cat. #11330-032), and 20 μg/ml PI (Sigma-Aldrich, Cat. # P4864)]. PI fluorescence was collected using 5-laser BD LSRFortessa flow cytometer, and data were analyzed with BD FACSDiva 8.0.2.

### 2.6 Cell viability and apoptosis assay

B16F10 (from ATCC) and 1014 (the kind gift of Lionel LaRue, Pasteur Institute) melanoma cells were grown in DMEM/F-12 + 10% fetal bovine serum(FBS) in a 37°C, 5%CO2 humidified incubator (ThermoFisher Heracell Vios 160i) until cells were at approximately 80-90% confluent in T150 flask (Corning Cat. #431465). Cells were trypsinized with 0.05% trypsin, 0.53 mM EDTA 1X (Corning Cat. #25-052-CI) for 5 minutes in a humidified CO2 incubator and then checked under the microscope to ensure that all cells had been released from the flask.

Cells were collected from the flask and counted by using a cell counter (Gibco Cell Countess II). Cells were then plated at 10,000 cells/well in each 96-well plate (Genesee Scientific Cat. #25-109MP and Thermoscientific Cat. #165305 [optical bottom plate with black base]) in triplicate using FluoroBrite DMEM (Gibco Cat. #A18967-01)+ 10% FBS (100μL). Cells were allowed to attach to plates overnight, and treatments were initiated the next day. The zero (0) concentration was a DMSO control diluted in the same manner as the highest concentration of the PF inhibitor. The treatments were as follows: palbociclib (palbo) alone at final concentrations of 100nM, 500nM,1μM, and 10μM, PF-07104091 inhibitor (PF) alone at final concentrations of 50nM,100nM, 500nM,1μM, and 10μM, and for the four concentrations of Pablo each of the five concentrations of PF inhibitor were added (ex. 100nM palbociclib +50nM PF inhibitor, 100nM palbociclib + 100nM PF inhibitor, 100nM palbociclib + 500nM PF inhibitor, 100nM palbociclib + 1μM PF inhibitor, and 100nM palbociclib + 10μM PF inhibitor). Treatment solutions were prepared at a 2X concentration of inhibitor in FluoroBrite DMEM media, with a final volume of 50μL. Removal of 50 μL of the original 100 μL of plated media was performed using a multichannel pipet and 50 μL of the treatments was added in triplicate wells following the template. Treatments were for 48, 72 or 144 hours. Cell viability was determined using the Cell Titer Blue assay (Promega, Cat. #G808A). Triplicate wells were set up with 100μL media containing no cells for background readings. The average of these three wells was subtracted from the reading of each treated well. The DMSO control wells were assigned a value of 100% viability, and a % viability was assigned to each treatment group and graphed based on this determination.

Apoptosis was determined by using the APO-One Homogeneous Caspase 3/7 assay (Promega, Cat. #G7791). Triplicate wells were set up with 100μL media with no cells for background readings. The average of these three wells were subtracted from the reading of each treated well. The amount of apoptosis is determined by the RFU reading from each well. These readings were averaged across the triplicate plates and graphed based on the RFU readings from each treatment ± standard deviation (SD). Statistical analysis was by ANOVA (Type III test). Additionally, we evaluated the RFU readings on a per cell basis to determine whether loss of cells contributed to reduction in the RFU readings per well.

### 2.7 Protein array

Mouse serum samples were prepared and examined in protein arrays using Mouse Antibody L308 Array Kit (308 proteins) (Cat. # AAM-BLG-1-4, RayBiotech), per the manufacturer’s protocol. A serum sample was taken from two B16F10 tumor-bearing mice in each treatment group for analysis. The glass chip was scanned on the Cy3 channel of a GenoPix 4000B scanner (Genopix 6.1, Molecular Devices, Sunnyvale, CA). For each spot, the net density was determined by subtracting the background.

### 2.8 Statistical analysis

Statistical analyses were performed using Prism software version 8.3.0 (GraphPad Software, RRID : SCR_002798). Data were summarized in figures displaying the mean ± SD. Treatment effects in standard two-group experiments were compared using a two-way ANOVA with unequal variances or the Wilcoxon rank-sum test. For Western blot data, a one-way ANOVA was used with unequal variances and Tukey’s multiple comparisons test. Where indicated, *p < 0.05; **p < 0.01; ***p<0.001; ****p<0.0001. For statistical analysis of the effects of targeted therapies on tumor volume, a mixed-effects model was used to account for the correlation among repeated measurements per mouse over time. Tumor volume was analyzed on a natural log scale to reduce heterogeneity. The mean tumor growth rates were estimated based on least-square means and compared using the Wald test. Pairwise comparisons were adjusted for p-values using the Holm correction.

A test for synergism between treatments was conducted, and a synergistic effect on tumor volume over time was defined as an effect from the drug combination (effect i) that exceeds the sum of the effects of each drug (effects a and b) (i.e., i>a+b). To evaluate this, a mixed-effects model including both individual and interaction effects was used to assess the impact of the drug combination on tumor volume over time. Model-based (least-squares) means were used to estimate average tumor growth for each treatment group. Synergism was evaluated using a multiple comparisons procedure within a generalized linear hypothesis testing framework, comparing the null hypothesis (i ≤ a + b) against the alternative (i > a + b). Standard residual diagnostics were also performed to validate model assumptions.

## 3 Results

### 3.1 Cell cycle CDK4/6 and CDK2 inhibitors differentially affect the cell viability and apoptosis of melanoma cells

**Figure 1:**
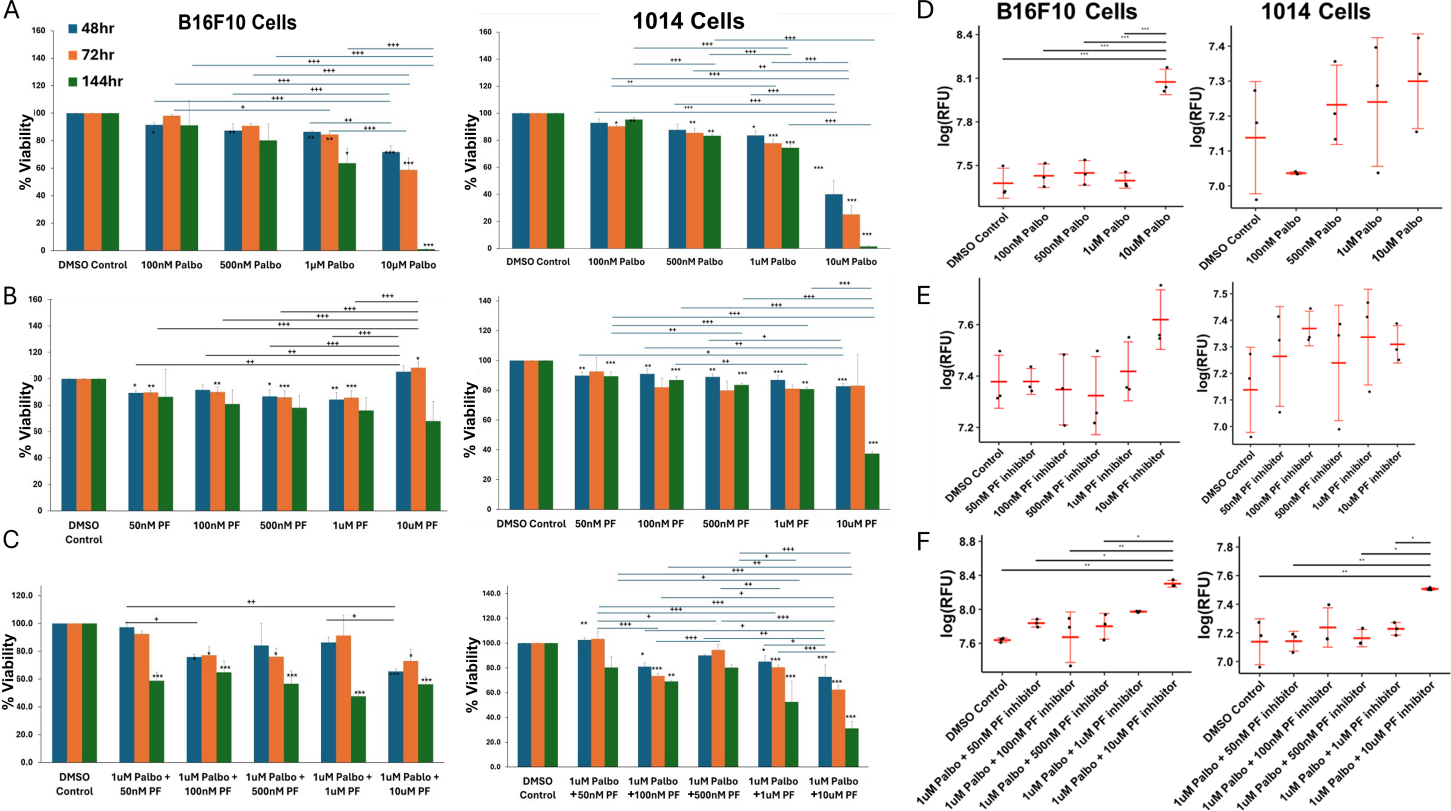
Cell cycle CDK4/6 and CDK2 inhibitors differentially affect melanoma cell viability and apoptosis. **(A)** effects of palbociclib or PF-07104091 on the *in vitro* viability/growth of B16-F10 melanoma cells over a concentration range of 100 nM to 10μM after 48h hour exposure; effect of PF-07104091 (PF-07104091 50 nM to 10 μM) on the viability/growth of B16F10 cells after 48h hour exposure; effect of 1μM palbociclib combined with concentrations of 50nM to 10μM of PF-07104091 on the viability/growth B16F10 melanoma cells after 48h hour exposure. **(B)** effects of palbociclib or PF-07104091 on the *in vitro* viability/growth of 1014 melanoma cells over a concentration range of 100 nM to 10 μM after 48h hour exposure; effect of PF-07104091 (PF-07104091 50nM to 10μM) on the viability/growth of 1014 cells after 48h hour exposure; effect of 1 μM palbociclib combined with concentrations of 50 nM to 10 μM of PF-07104091 on the viability/growth melanoma cells after 48 hour exposure. **(C)** palbociclib (5 or 10 μM) reduced the G1 to S-G2 transition of B16-F10 melanoma after a 24 hour exposure. **(D)** effects of palbociclib or PF-07104091 on the *in vitro* apoptosis of B16-F10 melanoma cells over a concentration range of 100nM to 10 μM after 48h hour exposure; effect of PF-07104091(50 nM to 10 μM) on the apoptosis of B16F10 cells after 48h hour exposure; effect of 1 μM palbociclib combined with concentrations of 50 nM to 10 μM of PF-07104091 on the viability/growth B16F10 melanoma cells after a 48 hour exposure. **(E)** effects of palbociclib or PF-07104091 on the *in vitro* apoptosis of 1014 melanoma cells over a concentration range of 100 nM to 10 μM after 48h hour exposure; effect of PF-07104091 (50 nM to 10 μM) on the viability/growth of 1014 cells after a 48h hour exposure **(F)**; effect of 1μM palbociclib combined with concentrations of 50 nM to 10 μM of PF-07104091 on the viability/growth melanoma cells after a 48 hour exposure. Data are shown as RFUs of caspase activity at each concentration of palbociclib. Adjusted p-values were analyzed using the Holm test to adjust for multiple comparisons. Adj. Sig. ***p<0.001, **p<0.01, and *p<0.05. The stars over the histogram indicate values that are significantly different from the DMSO control. The stars over the bars indicate the specific differences between treatment groups as indicated.

We first evaluated the effects of the CDK4/6 inhibitor palbociclib, the CDK2 inhibitor PF-07104091, or the combination of drugs on cell viability based on quantitation of cell titer blue staining. Cultured B16-F10 cells and 1014 cells were treated with palbociclib or PF-07104091 over a concentration range of 0 to 10 μM for 48, 72 or 144 hours. We observed a dose-dependent inhibition of cell viability in B16F10 (100 nM -10 μM) and 1014 (100 nM to 10 μM) in response to palbociclib over a 48, 72 and 144 hour time frame. However, the 1014 cells exhibited a greater reduction in viability at the 48- and 72-hour time points at a 10 μM concentration of palbociclib than the B16F10 cells (**Figure 1A**, **Supplementary Figure S1** **).** In contrast, at the 48-, 72- and 144-hour timepoints there was only a modest reduction in cell viability in response to PF-07104091 (50 nM to 10 μM) in both B16F10 and the 1014 cells showed a 60% inhibition in viability after 144 hours treatment with 10μM PF-07104091 (**Figure 1B**, **Supplementary Figure S1** **).** Interestingly, the addition of only 50 nM PF-07104091 to 1μM palbociclib significantly reduced the viability in B16F10 cells (p<0.001), while addition of 100 nM (p<0.01)or 1 μM (p<0.001) of PF-07104091 to 1 μM palbociclib significantly inhibited the viability of 1014 cells after 144 hours of treatment (**Figure 1C**, **Supplementary Figure S1** **).** To observe the induction of apoptosis of B16F10 and 1014 melanoma cells over time in response to palbociclib, we examined caspase 3/7 activity over 48 hours. Only modest induction of apoptosis occurred with 10 nM-1 μM concentrations of palbociclib in B16 melanoma cells, but 10 μM palbociclib induced a significant amount of apoptosis (**Figure 1D** **).** PF-07104091 did not significantly affect caspase 3/7 activity in B16F10 melanoma cells (**Figure 1E**). However, when combined with 1 μM palbociclib and increasing concentrations of PF-07104091, there was a significant increase in caspase 3/7 activity at the 10 μM concentration of PF-07104091 (**Figure 1F** **).** In contrast, 1014 cells exhibited highly variable levels of apoptosis which trended upward without showing a significant induction of caspase activity at the 48-hour timepoint to palbociclib or PF-07104091 (**Figures 1D, E** **),** though the combination of 1 μM palbociclib and 1 μM PF-07104091 as well as 1 μM palbociclib and 10μM PF-07104091 significantly increased apoptosis (**Figure 1F** **).** It is unclear why we did not detect increases in caspase activity in 1014 cells in response to palbociclib at the 48-hour timepoint, although we did observe a reduction in viability at this time point. However, when we analyzed RFU/cell, we did detect significant induction of APO-One activity with the 10 μM concentration of palbociclib at the 48-hour timepoint in 1014 cells. We speculate that the early loss of dying cells floating in the media may have contributed to the inability to capture a significant difference in the total RFU for caspase activity in each well of cells. At the 72- and 144-hour time points there were greater increases in caspase 3/7 activity in response to single treatment and combination treatments (**Supplementary Figure S1** **).** Notably, the combination of 10 μM palbociclib and 10 μM PF-07104091 resulted in the elimination of melanoma cell viability after 48 hours of drug treatment (**Supplementary Figure S1** **).**

### 3.2 CDK4/6 and CDK2 inhibitors modulate cell cycle and cyclin levels and reduce the hyperphosphorylation of Rb

It is known that for cells to transit from the G1 to S phase in the cell cycle, the tumor suppressor pRb needs to become hyperphosphorylated (29). This hyperphosphorylation is catalyzed by the complex formed by CDK4/6 and the cyclin group of related D cyclins of which cyclin D1 is a member), as well as by the CDK2/Cyclin complex. Upon CDK4/6 phosphorylation of pRb, it becomes partially inactivated, releasing the E2F transcription factors, which activate the E2F transcriptional program, including cyclin E1. Cyclin E1 binds to CDK2 to form an active complex that fully phosphorylates Rb, resulting in the full activation of the E2F transcriptional program and progression through the S-phase of the cell cycle. The transcription factor E2F is released, transcription of E2F regulated cell cycle genes, including Cyclin E1 ensues, and cells progress to S phase (30–32).

**Figure 2:**
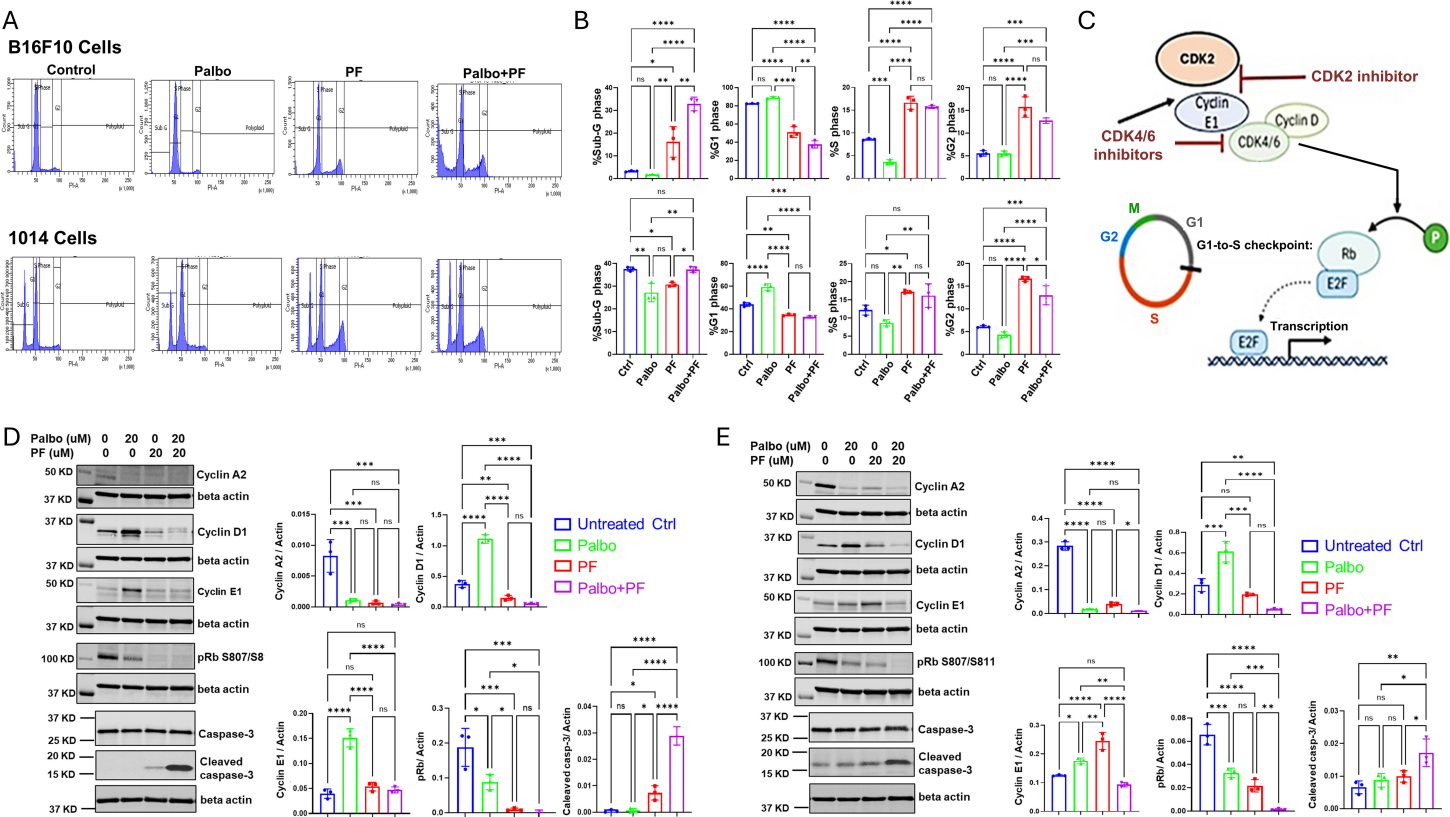
CDK4/6 and CDK2 inhibitors modulate cell cycle and cyclin levels and reduce the hyperphosphorylation of Rb in Braf-wild type melanoma. **(A)** representative flow cytometry cell cycle analysis for B16F10 and 1014 cells after treatment with control, palbociclib, PF-07104091, or the combination for 24 hours. **(B)** quantitation and statistical analysis of flow cytometry triplicate analysis of the effects of palbociclib, PF07104091, or their combination on cell cycle as compared to control. **(C)** diagram showing how the cascade of cyclin and pRb phosphorylation regulates the cell cycle and how cyclin E1 is up-regulated by palbociclib and down-regulated by the combined treatment of palbociclib and PF-07104091 in B16-F10 melanoma cells. **(D)** immunoblots and quantitative analysis of triplicate analysis of cell cycle proteins Cyclin A2, Cyclin D1, Cyclin E1, pRb, and cleaved caspase 3 in B16-F10 cells treated for 24 hours with either vehicle, palbociclib (20 μM), PF-07104091 (20 μM) or both inhibitors at 20 μM. **(E)** immunoblots and quantitative triplicate analysis of cell cycle proteins Cyclin A2, Cyclin D1, cyclin E1, pRB, and cleaved caspase 3 in 1014 cells treated with either vehicle, palbociclib (20 μM), PF-07104091 (20 μM) or both inhibitors at 20 μM. * p<0.05; ** p<0.01; *** p<0.001; **** p<0.0001; ns, non significant.

To evaluate the effects of CDK4/6 and CDK2 inhibitors on the cell cycle of B16F10 and 1014 melanoma cells, we chose a 24-hour treatment time to ensure capture of early and drug-specific effects. After examining a range of drug concentrations, we found that 20 μM concentrations of the drug, but not 10 μM concentrations, had significant effects on the cell cycle at the 24-hour time point. Therefore, subsequent experiments were performed with 20 μM concentrations of the drugs. We observed that 24-hour treatment of B16F10 cells with 20 µM palbociclib reduced the percentage of cells in S phase and had no significant effect on the percentage of cells in G1 or G2. At the same time, PF-07104091 increased the percentage of cells in S phase, decreased the percentage in G1 phase, and increased the percentage of cells in G2 and subG_0_ (dying cells) compared to untreated control. The combination of palbociclib and PF-07109091 decreased the percentage of cells in G1, increased the percentage in S and G2 and markedly increased the dying cells in subG_0_ compared to the untreated control (**Figures 2A, B**, upper panel). In 1014 cells the 24-hour, 20 μM treatment with palbociclib resulted in a decrease in sub-G_0_, an increase in G1, but no change in S or G2 phases as compared to control. PF-07104091 (20 μM) decreased the percentage of cells in sub G_0_ and G1 but increased the percentage of cells in S and G2 compared to control. The combination treatment decreased the percentage of cells in G1, had no effect on cells in S of sub G_0_ phase, and increased the percentage of cells in G2 as compared to control (**Figures 2A, B**, lower panel). A diagram showing the expected effects of CDK4/6 and CDK2 inhibitors on cell cycle proteins is shown in **Figure 2C**.

To evaluate the effects of CDK4/6 and CDK2 inhibitors on cyclin and cleaved caspase 3 protein levels we again utilized a 24-hour time period and treated cells with high concentrations of inhibitors (20 μM) to effectively maximize the ability to capture inhibitor-induced changes in cell cycle proteins by Western blot analysis. In B16F10 melanoma cells, palbociclib decreased protein levels of cyclin A2 but increased cyclin D1 and cyclin E1 protein levels (**Figure 2D**). The CDK2 inhibitor decreased protein levels of cyclin A2 and cyclin D1 and had no significant effect on cyclin E1 protein levels. Combined treatment of B16-F10 cells with palbociclib (20μM) and the CDK2 inhibitor PF-07104091 (20μM) resulted in a reduction of both cyclin A2 and cyclin D1, reversed the palbociclib induction of cyclin E1, and strongly induced cleaved caspase 3 levels (**Figure 2D**). Hyperphosphorylation of Rb(S807/811) was reduced by treatment with palbociclib (20μM) and was blocked entirely by PF-07104091 (20 μM), with the combination of palbociclib and PF-07104091 not having an effect greater than PF-07104091 alone (**Figure 2D**).

In 1014 cells, palbociclib (20μM) significantly reduced protein levels of cyclin A2, increased cyclin D1 levels and cyclin E1 levels, and reduced hyperphosphorylation of Rb (S807/S811). PF-07104091 (20μM) decreased cyclin A2 levels, had no significant effect on cyclin D1 levels, increased cyclin E1 levels and cleaved caspase 3 and reduced hyperphosphorylation of Rb (S807/S811). The combination treatment with palbociclib (20μM) and PF-07104091 (20μM) significantly reduced cyclin A2, reduced cyclin D1, and blocked phosphorylation of Rb (S807/S811). It reversed the elevations of cyclin E1 by each agent alone, restoring cyclin E1 levels to the control level (**Figure 2E** **).** The combination treatment also strongly induced cleavage of caspase 3 (**Figure 2D**).

The individual inhibitors and their combination resulted in a decrease in cyclin A2 levels (**Figures 2D, E**), which is similar to a previous report by Arora et al. in breast cancer cells. However, the concentration of the drug used here was higher (24). It is curious that, despite nearly complete inhibition of hyperphosphorylation of Rb S807/811 in response to 20 μM of PF-07104091 in B16F10 melanoma cells (**Figure 2D** **),** treatment with 10 μM of PF-07104091 for up to 48 hours had only a modest effect on cell viability (**Figure 1A**). In contrast, the 1014 cells were less sensitive to PF-07104091 regarding the reduction in Rb phosphorylation. However, the 24-hour treatment with a combination of palbociclib (20 μM) and PF-07104091 (20 μM) effectively blocked Rb807/811 phosphorylation and induced cleaved caspase 3 in both B16F10 and 1014 cells.

### 3.3 The CXCR1/2 antagonist, SX-682, affects the growth-inhibitory effect of palbociclib and anti-tumor immunity *in vivo*

**Figure 3:**
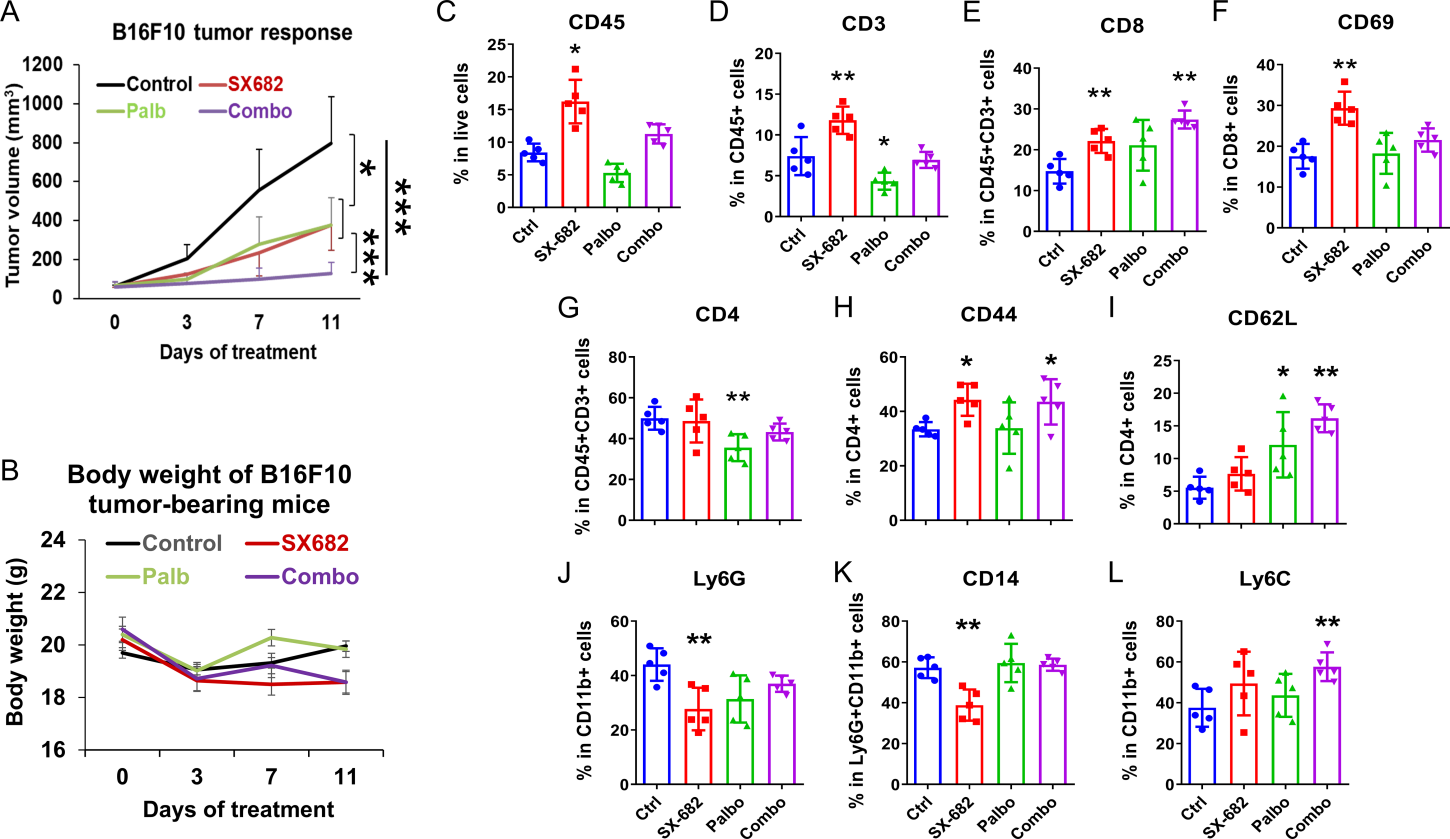
The CXCR1/2 antagonist, SX-682, affects the growth-inhibitory effect of palbociclib and increases anti-tumor immunity *in vivo*. **(A)** the effect of palbociclib dosing (100 mg/kg treatment), SX-682 chow, or the combination on body weight or **(B)** tumor growth of B16-F10 tumor-bearing C57Bl/6 mice. **(C–L)** the effect of palbociclib, SX-682, or the combination on various immune populations in the TME of B16-F10 tumor-bearing C57Bl/6 mice. *p<0.05, **p<0.01, ***p<0.001.

To evaluate the hypothesis that the previously demonstrated anti-tumor effects of palbociclib on the memory CD8 T-cell pool (13) would be enhanced by co-treatment with SX-682, which has been shown to reduce the recruitment of myeloid-derived suppressor cells (MDSCs) into the TME, we compared the effects of palbociclib alone versus palbociclib in combination with the CXCR1/2 antagonist SX-682 on the tumor growth of B16-F10 melanoma in C57BL/6 mice. Tumor-bearing mice (5 mice/group) were treated with 100 mg/kg per day palbociclib HCL alone (5 days/week) or combined with chow containing SX-682. Because these tumors grow very rapidly, treatment was initiated when the tumor diameter was ~5mm yielding a tumor volume of ~125mm^3^ to ensure that here was adequate time to monitor response to drug before control mice had to be euthanized due to tumor burden. Moreover, this ensures that at the end point, the tumors have not developed sufficient necrosis to limit the evaluation of the TME by flow cytometry. The anti-tumor effect of palbociclib was enhanced with the addition of SX-682 (**Figure 3A**), but synergism between the two treatments was not detected (**Supplementary Figure S3A**). All treatment groups tolerated the treatment for eleven days without significant loss of body weight (**Figure 3B**).

To examine the immune response to palbociclib and/or SX-682 treatment in the TME of tumors from **Figure 3A**, a single-cell tumor suspension was prepared, cells were stained with fluorescent conjugated antibodies and analyzed by flow cytometry. In comparison with vehicle controls, tumor-bearing mice fed with SX-682 chow showed an increase in the TME of CD45+ total tumor-infiltrated leukocytes (**Figure 3C**), including CD3+CD45+ T cells (**Figure 3D**) in the TME, an increase in CD8+ T cells (**Figure 3E** **),** an increase in CD69+ activated CD8+ T cells (**Figure 3F**), and an increase in the percentage of CD4+CD44+ T cells (**Figure 3I**). SX-682 also reduced the percentage of Ly6G+ CD11b+ and CD14+Ly6G myeloid cells (**Figures 3J, K**) in the TME. In contrast, palbociclib treatment reduced the percentage of CD3+CD45+ T cells (**Figure 3D**), decreased the CD4+CD3+ T cells (**Figure 3G**) in the TME as compared to control. The combination treatment (palbociclib + SX-682) compared to control exhibited a normalization of the effect of SX-682 on the percentage of CD3+ T cells in the tumor (**Figure 3D** **).** The combination treatment also increased the percentage of CD3+CD8+ T cells (**Figure 3E**), increased the percentage of CD44+CD4+ T cells (**Figure 3H** **),** increased the percentage of CD4+CD62L+ T cells (**Figure 3I**), and increased the percentage of CD11b+Ly6C+ monocytes (**Figure 3L**) compared to control. In some instances, the positive effects of the SX-682 chow on the anti-tumor immune environment were thus overridden by the addition of palbociclib (i.e., loss of reduction in Ly6GCD11b+ cells, loss of reduction in CD14+Ly6G+ cells, increase in Ly6C+CD11b+ cells, loss of increase in CD69+ CD8+ T cells, and reduction in total CD45+ cells and loss of increase in CD45+ CD3+ T cells compared to SX-682). However, the percentage of CD8+ T cells remained elevated in tumors treated with both SX-682 and palbociclib. The effects of combined therapies were additive, but not synergistic in B16-F10 melanoma tumors (**Supplementary Figure S2A**). Palbociclib appears to be exerting its major effect by slowing the movement of tumor cells through the cell cycle, thus slowing tumor growth. In contrast, SX-682 affects tumor growth (27) and produces a more anti-tumor immune environment characterized by increased CD8+ T cells and activated CD69+CD8+ T cells (**Figures 3E, F**).

### 3.4 SX-682 treatment tends to increase serum levels of cytokines involved in T cell response in B16-F10 tumor-bearing mice

**Figure 4:**
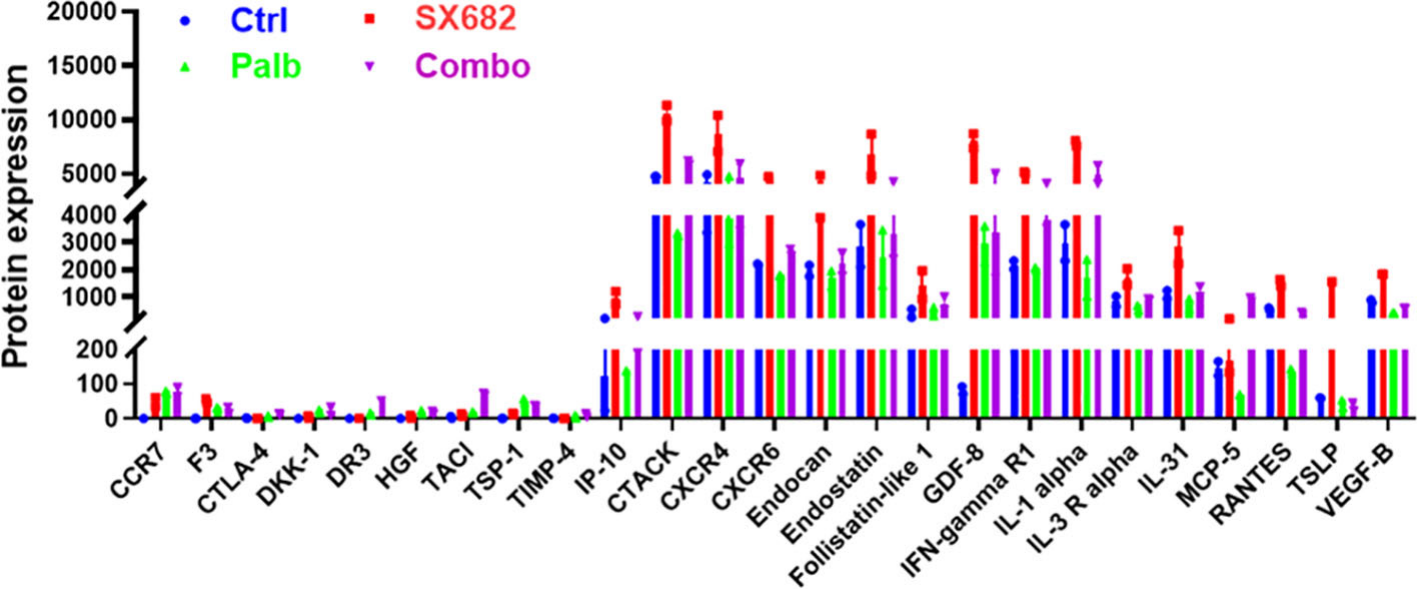
SX-682 treatment results in a trend toward increased serum levels of cytokines involved in T cell response in B16-F10 tumor-bearing mice. Comparison of protein levels in serum from B16F10 mice treated with SX-682, palbociclib, or the combination, normalized to mice in the vehicle control group.

A protein array was performed on the sera from B16F10 tumor-bearing C57Bl/6 mice at the endpoint of the tumor growth assay to define the values for 308 cytokines or receptors in each of the four treatment groups (**Figure 4**). Results showed that the mice in the SX-682 treatment group exhibited trends toward increased levels of IP-10 (CXCL10), CTACK, CXCR4, CXCR6, Endocan, Endostatin, GDF-8, IFNγR1, IL-1α, IL-31, MCP-5 and TSLP compared to the control group. These cytokine array data demonstrate that treatment with the CXCR1/2 antagonist elevates factors associated with T cell activation and recruitment [IP-10 (CXCL10), CTAK, CXCR4, CXCR6, IFNγR1, IL-1α, TLSP] over that with the CDK4/6 inhibitor. Both SX-682 and palbociclib inhibitors suppressed amphiregulin. IL-1α, endostatin, GDF8, and IFNγR1 also trended upward in the serum of mice treated with the combination of SX-682 + palbociclib. Palbociclib did not increase the values of the cytokines assayed here over control, except GDF-8. Since the data represent duplicate values, statistically significant differences cannot be determined. Altogether, these data reinforce prior published data showing that SX-682 treatment increases the recruitment of T cells and activated T cells into the TME (27).

### 3.5 Palbociclib, SX-682, and the combination of both drugs inhibit NRAS mutant 1014 melanoma tumor growth and result in a stronger anti-tumor immune microenvironment

**Figure 5:**
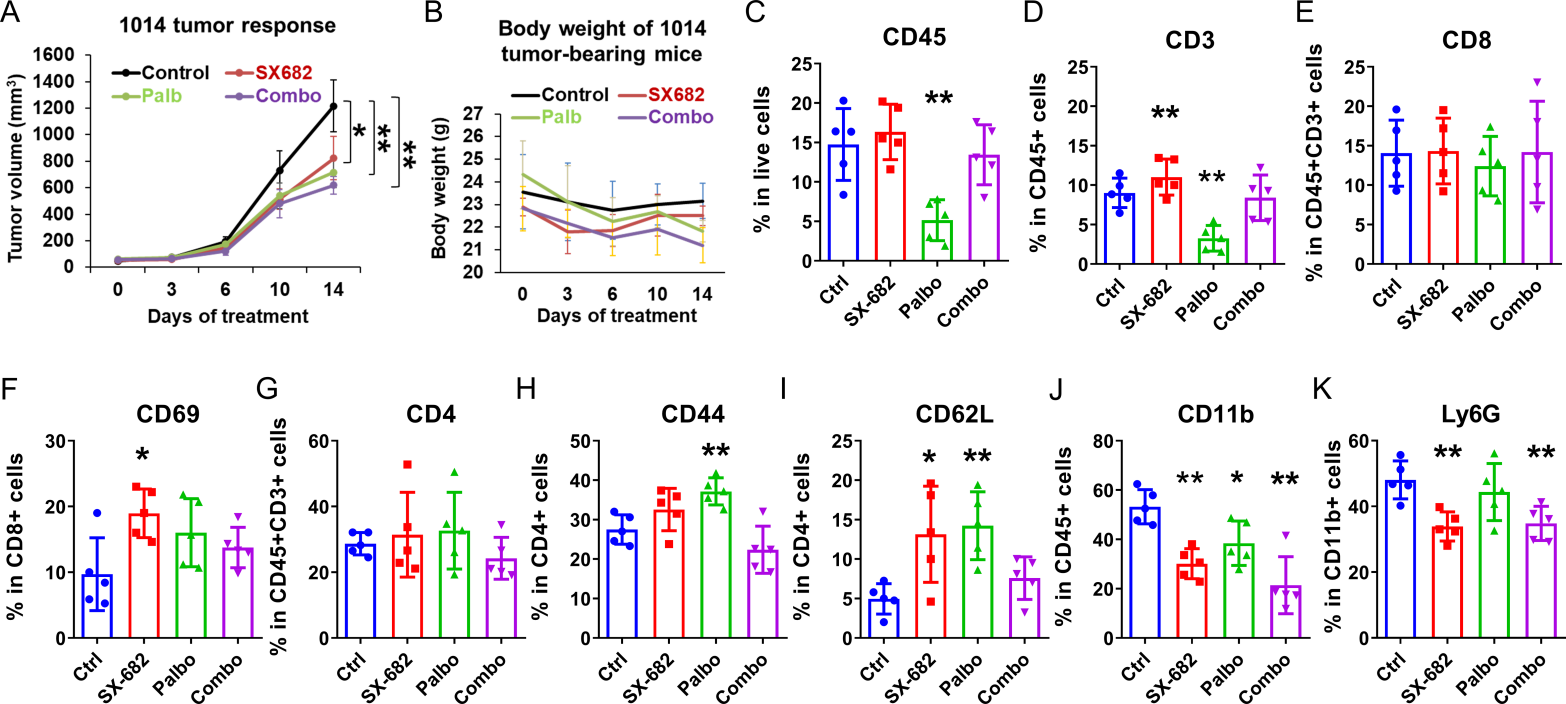
Palbociclib, SX-682, and the combination of both drugs inhibit Nras mutant 1014 melanoma tumor growth and result in a stronger anti-tumor immune microenvironment. The effect of palbociclib dosing (100 mg/kg treatment), SX-682 chow, or the combination on **(A)** tumor growth, or **(B)** body weight of C57Bl/6 mice bearing NRAS^mut^ 1014 melanoma tumors. **(C–K)** profile of immune cell populations in the TME of NRAS^mut^ 1014 tumor-bearing mice receiving treatment with palbociclib (100 mg/kg), SX-682 inhibitor containing chow, or the combination of treatments. *p ≤ 0.05, **p ≤ 0.01.

Experiments evaluating the effect of SX-682, palbociclib, or the combination of inhibitors were also conducted in C57Bl/6 mice bearing 1014 NRAS mutant (NRAS^mut^) melanoma xenografts over a treatment period of two weeks. Palbociclib significantly inhibited tumor growth (p<0.01), as did SX-682 (p<0.05). However, the effect of the combination treatment on inhibiting tumor growth was not greater than that of either treatment alone (**Figure 5A**). Individual therapies and the combination treatment did not result in a body weight reduction of greater than 10% over the treatment period (**Figure 5B**). Palbociclib reduced the percentage of CD45+ cells and CD3+CD45+ T cells in the tumors, but this effect was reversed by the combination with SX-682 treatment (**Figures 5C, D**). While neither treatment increased the percentage of CD3+ T cells that were CD8+ T cells, SX-682 chow increased the percentage of CD69+ activated CD8+ T cells (**Figures 5E, F**). Also, neither treatment affected the percentage of CD3 T cells that were CD4+. Both palbociclib and SX-682 increased the effector memory (CD44+) and naive (CD62L+) CD4+ T cells (**Figures 5G–I**), but the combination treatment was similar to the control treatment (**Figure 5I**). SX-682 chow, palbociclib alone, and the combination of both SX-682 and palbociclib reduced the percentage of CD11b cells (**Figure 5J**). The percentage of CD11b+Ly6G+ granulocytes, presumably granulocytic MDSCs (gMDSCs), was only reduced by SX-682 (**Figure 5K**). The lack of an additive effect of palbociclib and SX682 on the growth of the 1014 cells is consistent with the palbociclib- mediated reduction of immune cells in the TME. The palbociclib inhibition of Rb hyperphosphorylation was equivalent in both B16F10 and 1014 cells. These data suggest that the increased recruitment of CD8+ T and CD4+ T cells in palbociclib + SX-682-treated B16F10 tumors but not in 1014 tumors may be associated with the observation that the 1014 tumors are less growth-inhibited by palbociclib plus SX-682 than the B16F10 tumors. Alternatively, activation of the NRAS pathway in 1014 cells may result in production of factors that override the inhibition of CDK4/6 and CXCR2.

### 3.6 The addition of a CDK2 inhibitor (PF07104091) to the CDK4/6 inhibitor palbociclib and the CXCR2 inhibitor SX-682 improves the anti-tumor response in BRAF wild-type melanoma

**Figure 6:**
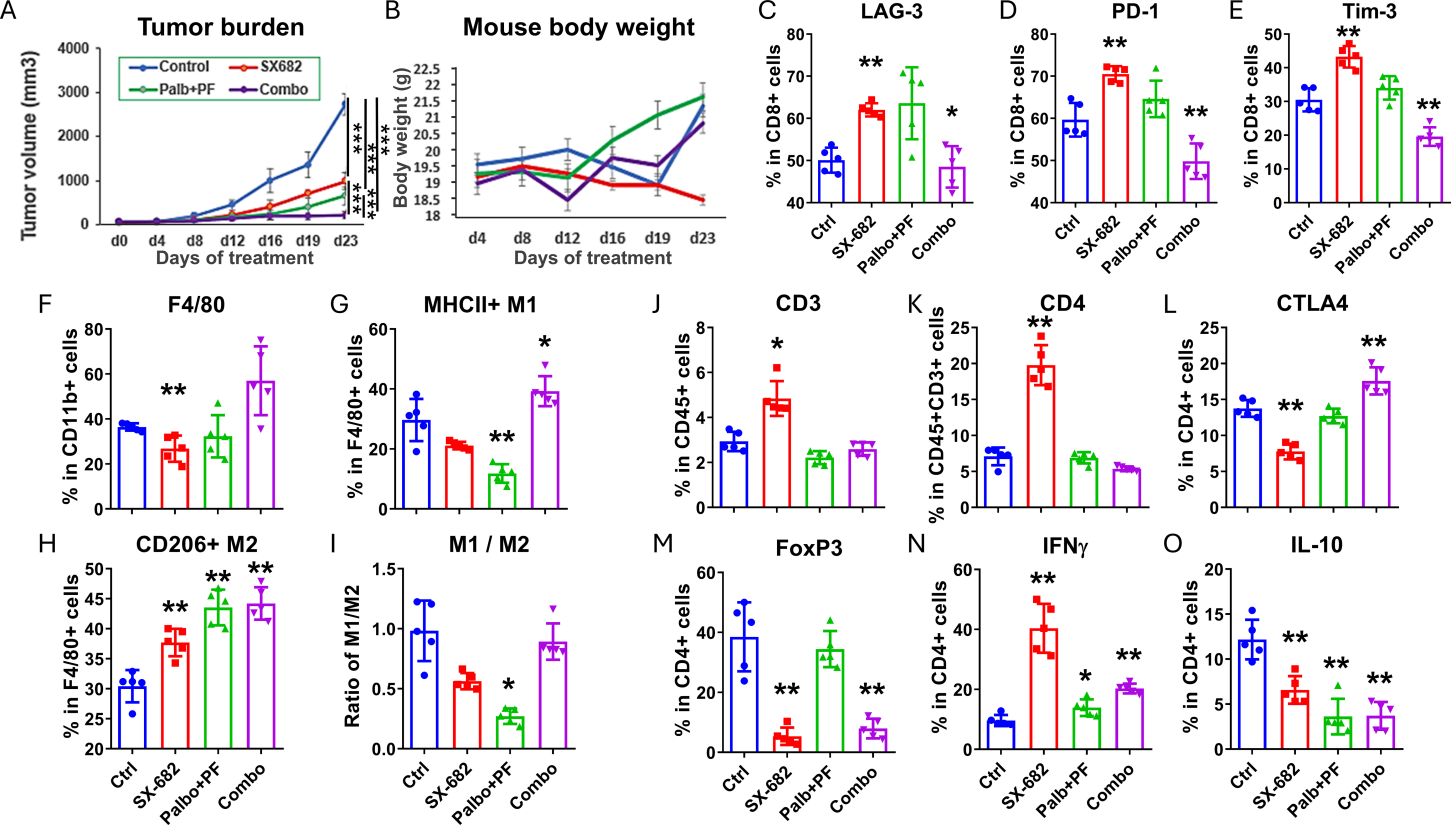
The addition of a CDK2 inhibitor (PF07104091) to the CDK4/6 inhibitor palbociclib and the CXCR2 inhibitor SX-682 improves the anti-tumor response in BRAF wild-type melanoma. **(A)** the effect of the following treatments on the tumor growth of B16F10 tumor xenografts growing in C57Bl/6 mice for four weeks: combination palbociclib (100 mg/kg) and PF 07104091 (50 mg/kg), SX-682 chow, or the combination of all three inhibitors. **(B)** the effect of the above treatments on B16F10 mouse weight in C57Bl/6 mice over 23 days. **(C–O)** the effect of palbociclib + PF07104091, SX-682, or the combination on various immune populations in the TME of C57Bl/6 mice bearing B16F10 tumors. *p ≤ 0.05, **p ≤ 0.01; ***p ≤ 0.001.

When C57Bl/6 mice (10 mice/group) bearing B16-F10 tumors (~5mm diameter) were exposed to a daily dose of 100 mg/kg palbociclib, 50 mg/kg of the CDK2 inhibitor, PF-07104091, with control chow or SX-682 chow, the toxicity of palbociclib plus PF-07104091 or the triple therapy was acceptable and anti-tumor efficacy of the triple combination with SX-682 was increased relative CDK4/6 plus CDK2 inhibition or SX-682 alone (**Figures 6A, B**), resulting in a failure of tumors to grow. The effect of the triple combination treatment on tumor growth was additive, but not synergistic (**Supplementary Figure S2B** **).**

Analysis of the immune cells in the TME of B16-F10 (**Figures 6C-O**) melanoma tumors treated with SX-682 alone increased the CD3+ T cells. Palbociclib+ PF-07104091 reduced the CD3+ T cells, increased IFNγ+ CD4+ T cells, but reduced IL-10 + CD4+ T cells. This CDK inhibitor combination was associated with a decrease in F4/80+MHCII+ M1-like macrophages and an increase in CD206+F4/80+ M2-like macrophages. The triple combination of CDK4/6, CDK2, and CXCR1/2 antagonists revealed an increase in CD4+CTLA4+ T cells, an increase in IFNγ+ CD4+ T cells, but a reduction in Tregs (CD4+FOXp3+), CD8+PD-1+ T cells, CD8+Tim-3+ T cells, and IL-10+ CD4+ T cells. These data indicate that there is a reduction in the exhaustion status of CD8+ T cells, an increase in CD4+ T cells, and a decrease in Tregs with the triple combination inhibitor treatment. Altogether, these data support combining inhibitors of CDK4/6, CDK2, and CXCR1,2 for the treatment of BRAF wild-type melanoma. The CDK inhibitors work together to inhibit Rb phosphorylation while the CXCR1,2 antagonist creates a more anti-tumor immune microenvironment.

**Figure 7:**
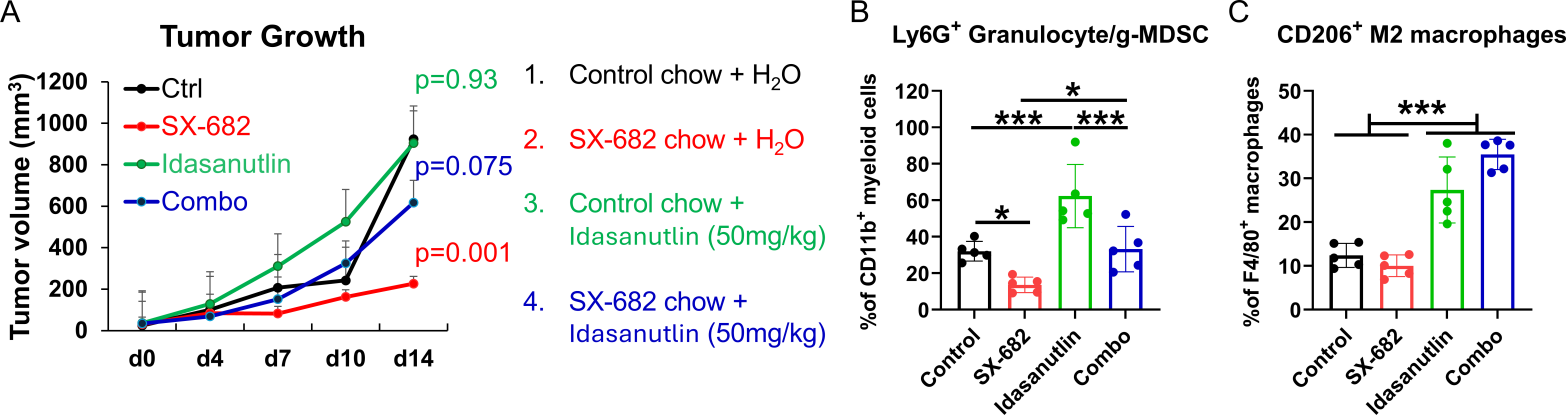
The MDM2 antagonist idasanutlin ablates the tumor growth inhibitory and anti-tumor immunity response to SX-682. **(A)** C57Bl/6 mice bearing B16F10 tumors were placed on chow containing SX-682 or control chow at the time tumors reached a diameter of 5mm. Mice were grouped into four groups: those that received control chow + vehicle, those that received SX-682 chow + vehicle, those receiving control chow and idasanutlin (50mg/kg), and those on SX-682 chow that also received idasanutlin (50mg/kg) by oral gavage. Treatments and measurements continued for fourteen days, after which time the mice were euthanized, and tumors **(B)** and blood **(C)** were collected for isolation and flow cytometry to characterize the immune cells. * p<0.05; *** p<0.001.

We have previously demonstrated in preclinical models that CDK4/6 inhibitors, when combined with MDM2 inhibition, can effectively inhibit melanoma tumor growth, and that resistance to CDK4/6 inhibition can be overcome through the targeted deletion of CDK2 (17). However, MDM2 inhibitors are mostly effective only in p53^WT^ tumors, and they have been shown to induce thrombocytopenia, neutropenia, and other toxicities, though several recent clinical trials continue to ‘fine-tune’ these inhibitors and combine them with other appropriate therapies for further clinical development (33–36). To determine how an MDM2 inhibitor might affect the response to SX-682 in melanoma, we performed experiments combining the MDM2 inhibitor, idasanutlin (50mg/kg), with SX-682 in B16F10 melanoma and we observed that idasanutlin reduced the effect of SX-682 on tumor growth inhibition (**Figure 7A**) and reversed the inhibitory effects of SX-682 on the Ly6G+CD11b+ granulocytic MDSCs (**Figure 7B**) and also increased the CD206+ M2 macrophages in the blood (**Figure 7C**). Thus, inclusion of an MDM2 inhibitor with treatment regimens that include the CXCR2 antagonist SX-682 is not advised. Our results show that optimal inhibition of tumor growth in the B16F10 melanoma model can be obtained with co-inhibition of CDK4/6 and CDK2 along with SX-682.

## 4 Discussion

Key to the process of cancer development is the failure to control cell proliferation, often resulting from an impaired regulation of proteins involved in cell cycle progression, particularly the constitutive activation of the cyclin-dependent kinases (CDKs) (37). Upon mitogenic stimulation, CDK4/6 hyperphosphorylates the tumor suppressor Rb, releasing the transcription factor E2F to drive expression of cyclin E, resulting in the elevation of this protein at the late restriction point of the G1 phase (38). When cyclin E binds to CDK2, and the active complex that also hyperphosphorylates Rb and numerous other substrates, controlling essential cellular processes, including the initiation of DNA replication and regulation of histone biosynthesis. Phosphorylated cyclin E protein is degraded by the SCF(Fbw7) ubiquitin ligase complex, thus eliminating cyclin E/CDK2 activity (39–41). A high level of cyclin E protein is associated with poor prognosis, reduced survival, and therapy resistance in cancer patients (40). Moreover, the overexpression of cyclin E has been proposed as a potential mechanism of resistance to CDK4/6 inhibitors (15, 42–44). Dysregulation of these kinases is a major contributor to endocrine therapy resistance in breast cancer (11).

CDK4/6 inhibitors induce cell cycle arrest in Rb protein (pRb)-competent cells by blocking the hyperphosphorylation of Rb by CDK4/6 (37, 45). Although early CDK4/6 inhibitors were quite toxic, the development of more selective CDK4/6 inhibitors, such as ribociclib, abemaciclib and palbociclib, has led to improved efficacy and reduced adverse events during the treatment of tumors that are driven by CDK4/6 pathway activation. Preliminary evidence showed promising activity in melanoma (17, 46) and improved progression-free survival with tolerable toxicity in patients with advanced-stage estrogen receptor (ER)-positive breast cancer, though resistance is an issue (42). Factors involved in acquired resistance to CDK4/6 inhibitors are being identified and new approaches to overcome this resistance are being developed. Biomarkers have been characterized that identify solid tumors that will not benefit from treatment with CDK4/6 inhibitors, such as loss of pRb (37).

The main objective of the study described in this report is to develop new approaches to improve the efficacy of CDK4/6 inhibitors for the treatment of melanoma. Our study design was informed by data comparing the clinical pharmacokinetics and pharmacodynamics of palbociclib (47–49). It is important to note that the GI50 concentrations of palbociclib (concentration of drug required to inhibit growth of 50% of the cells) required for the inhibition of melanoma cell growth here in B16F10 and 1014 cells is high (~10 μM) as compared to the reported GI50 of 100 nM to 1 μM for breast cancer cells. However, a wide range for the GI50 for melanoma cell lines has been observed previously by other groups, ranging from 30nM to 9 (16, 50, 51). The effective serum concentration of palbociclib delivered at a dose of 100 mg/kg body weight is between 3 and 5 μg/ml. Here we had a maximal response at 10 μM concentrations of palbociclib which is the equivalent of 4.473 μg/ml of palbociclib. There are many factors that affect the GI50, including genetic and phenotypic differences, culture conditions, time of exposure to the drug, cell density, genetic mutations and biomarkers, metabolic factors, prior drug treatment, and experimental techniques. Our data show the *in vitro* activity of palbociclib to be comparable to that reported by others for melanoma therapy.

The stability of cyclin D1 is regulated by Thr286 phosphorylation by GSK3β, and the stability of cyclin E1 is regulated by Thr380 phosphorylation of cyclin E1 by CDK2. These phosphorylation events allow cyclin ubiquitination by SCF(Fbx4/αB-crystallin) E3 ubiquitin ligase for cyclin D1 (52) and SCF(Fbw7) E3 for cyclin E1 (53), followed by degradation. We observed that palbociclib (20μM) alone induced cyclin D1 and cyclin E1 in B16F10 and 1014 cells, but in combination with the CDK2 inhibitor PF-07104091 (20 μM), this induction did not occur. The palbociclib increase in cyclin D1 levels in B16F10 and 1014 cells and the PF-07104091 increase in cyclin E1 in 1014 cells indicates that either the GSK2B and CDK2 phosphorylation may not be occurring, or the E3 ubiquitin ligase activity is deficient for these cyclins since the hyperphosphorylation of RB is inhibited by treatment with palbociclib and/or PF-07104091 with the combination treatment completely blocking phosphorylation of Rb on S807/S811. The increased cyclin E1 levels in 1014 cells in response to inhibition of CDK2 would be expected if CDK2 inhibition resulted in inability to phosphorylate cyclin E1 to target it for degradation. However, we did not observe a PF-07104091 elevation of cyclin E1 in B16F10 cells. In a separate study, low-dose CDK2 inhibition with PF-3600 resulted in a rebound phenotype that could be overcome with co-inhibition of both CDK4/6 and CDK2, or with high-dose PF-3600 (24). In 1014 melanoma cells, even with high-dose CDK2 inhibition, addition of the CDK4/6 inhibitor was required to suppress cyclin E1 levels. Clearly, the response to these inhibitors varies among different cell lines depending on the activity of a spectrum of proteins involved in regulating the cell cycle.

The data from the analysis of the effects of palbociclib, PF-07104091, and the combination of drugs on the progression through cell cycle were surprising. While we expected that palbociclib would reduce the percentage of cells in S phase and that PF-07104091 alone would increase the S phase percentage (24), we did not expect to see an increase in cells in G2, or a reduction of cells in G1 in response to the CDK2 inhibitor. These data suggest that in melanoma cells, the CDK2 inhibitor may impinge on progression from G2 to M phase, and this has not been previously reported. The effects of PF-07104091 and the combination with palbociclib on increasing the percentage of cells in subG_0_ could indicate that these drugs are inducing cell death in B16F10 cells, but not the more resistant 1014 cells. However, the combination of both inhibitors effectively induces apoptosis, reduces cell viability, and results in loss of hyperphosphorylation of Rb at S807/S811.

While CDK4/6 inhibitors have been effectively combined with ICI for the treatment of breast cancer, this combination with or without CDK2 inhibition has not yet been demonstrated to be clinically effective for metastatic melanoma. However, it has been demonstrated that CDK4/6 activity drives ICI resistance in RB competent immune cold melanoma tumor but when mouse models of these resistant melanoma tumors are treated first with ICI followed by CDK4/6 inhibition plus ICI, the resistance program is overcome and tumor growth is suppressed, demonstrating that order of delivery of the therapy is important.

In this study, we show that palbociclib is somewhat immune suppressive based upon its decrease in CD45+ cells, CD3+CD45+ T cells, CD4+CD3+ cells and CD11b+CD45+ cells, though it increased the CD44+CD4+ and CD62L+CD4+ T cells. In contrast, SX-682 blockade of CXCR1/2 in B16F10 tumors resulted in increased CD45+ immune cells, increased intratumoral CD8+T cells and CD69+CD8+ T cells, increased CD44+ CD4+ T cells and CD62L+ CD4+ T cells, and reduced CD11b+CD45+ cells, Ly6G+CD11b+ and CD14+Ly6G+ myeloid cells (**Figure 3C** **).** The combination of SX682 and palbociclib inhibited B16F10 melanoma tumor growth, increased the percentage of CD45+ cells, CD3+CD8+ T-cells, CD4+CD44+ T cells, and CD62L+CD4+ T cells. For myeloid cell effects, the combination decreased the percentage of CD11b+CD45+ cells and increased the Ly6c+CD11b+ cells. Analysis of data from a serum protein array indicated that mice fed SX-682 chow exhibited a trend toward higher levels of certain inflammatory cytokines involved in regulating T cells.

The effect of SX-682 or palbociclib individually on the growth of 1014 cells was equivocal to that for B16F10 melanoma cells (~50% inhibition). However, the effect if the combination therapy on the growth of 1014 NRAS^mut^ melanoma xenografts was less than that observed with the B16F10 xenografts, though SX-682 increased the CD69+CD8+ activated T cells and decreased the CD11b+CD45+ myeloid cells, including Ly6G+CD11b+ granulocytes (and/or gMDSCs) in 1014 tumors similar to that observed in the B16F10 tumors. Palbociclib increased the CD4+CD25^high^ T regulatory cells and the CD4+CD44+ effector memory T cells. Both palbociclib and SX-682 as single agents increased the CD4+CD62L+ central memory T cells, but the combination treatment eliminated this increase. This failure of the combined treatment with palbociclib and SX-682 to increase CD3+CD8+ T-cells, and CD62L+CD4+ T cells in 1014 tumors could explain in part the reduced response to SX-682 + palbociclib in 1014 tumors versus B16F10 tumors. As observed with SX-682 treatment, the combination treatment reduced the percentage of CD11b cells and the percentage of CD11b+ myeloid cells, including CD11b+Ly6G+ cells in both B16F10 and 1014 tumors, presumably gMDSCs.

In B16F10 tumors, addition of SX-682 containing chow to treatment with palbociclib + PF-07104091 blocked tumor growth and this was accompanied by reduced the percentage of CD8+ T cells, but also reduction in LAG-3+CD8+ T cells, TIM-3+ CD8+ T cells, PD-L1+ CD8+ T cells, Foxp3+CD4+ T cells, and IL-10+ CD4+ T cells in the TME, suggesting the CD8+ T cells were less exhausted compared to those in the control TME. In contrast, there was an increase in the CD4+ CTLA4+ T cells with the combined therapy. The combination treatment increased IFNγ+ CD4+ T cells, as well as M1-like and M2-like macrophages. Thus, addition of PF-07104091 to the SX-682 and palbociclib treatment was highly effective in inhibiting tumor growth and producing a more anti-tumor TME. Similar results would be expected for 1014 tumors, though the response would be expected to be somewhat reduced in comparison to B16F10 tumors, based on the more suppressive effect of palbociclib on the population of T cells in the TME.

A limitation of our study is that only two melanoma cell lines were evaluated in the study, BRAF^WT^/NRAS^WT^ B16F10 and NRAS^Q61R^ mutant 1014 cells. We concentrated on melanoma lines that do not have a mutation in BRAF, since there are adequate second-line therapies available for BRAF mutant melanoma patients, but not for BRAF wild-type melanoma patients. One might argue that the B16F10 model may not fully represent the BRAF/RAS^WT^ melanomas that usually harbor NF1 mutation or deletion, KIT mutation, or amplification of cyclin D. Certainly extension of the study to more representative murine models could provide additional information as to how various genetic modifications affect response to CDK4/6 and CDK2 inhibitors. On that note, we have previously examined the response of seven human melanoma cell lines and five melanoma patient-derived xenografts to treatment with a CDK4/6 inhibitor alone or in combination with an MDM2 inhibitor (17). We also demonstrated that blocking CDK2 activity enhanced the response to CDK4/6 inhibitors in these melanoma models. With SX-682 currently in clinical trials for the treatment of metastatic melanoma in combination with anti-PD1 in instances where there is resistance to ICI therapy, we propose that an alternative option may be to treat ICI-resistant BRAF wild-type melanoma with the combination of CDK4/6, CDK2, and CXCR2 antagonists.

## 5 Conclusions

Altogether, these data suggest that the addition of the CDK2 inhibitor to CDK4/6 and CXCR1/2 inhibitors not only reduces melanoma tumor cell viability and tumor growth more effectively in BRAF^WT^NRAS^WT^ melanoma cells but also results in a less immunosuppressive tumor immune microenvironment. This study provides significant information for the design of future clinical trials for ICI-resistant melanomas without BRAF mutation.

## Data Availability

The original contributions presented in the study are included in the article/**Supplementary Material**. Further inquiries can be directed to the corresponding authors.
