# Supplementary figures and images for: Combined treatment with CDK4/6, CDK2, and CXCR1/2 inhibitors effectively halts the growth of BRAF wild-type melanoma tumors
Source: Front Oncol. 2025 Aug 19;15:1609735. doi: 10.3389/fonc.2025.1609735 (PMC12402945; doi:10.3389/fonc.2025.1609735)

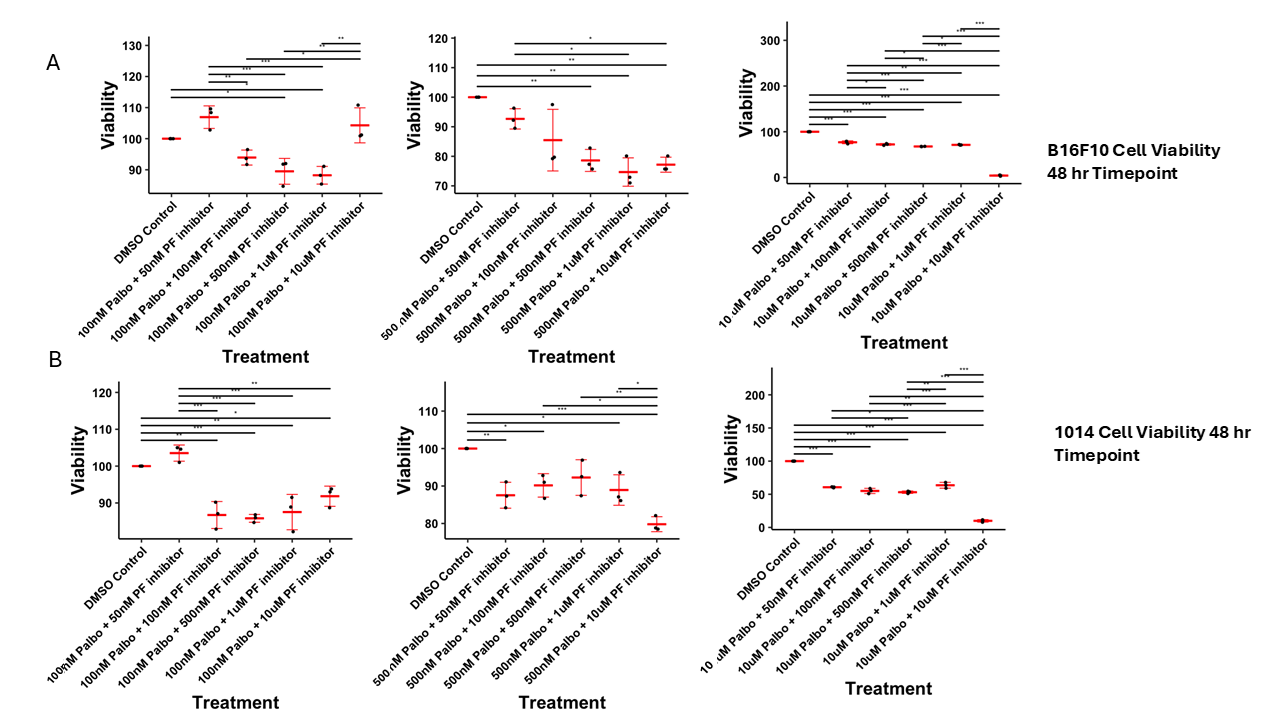

Supplement: Supplementary file 1 [file Image1.tif]

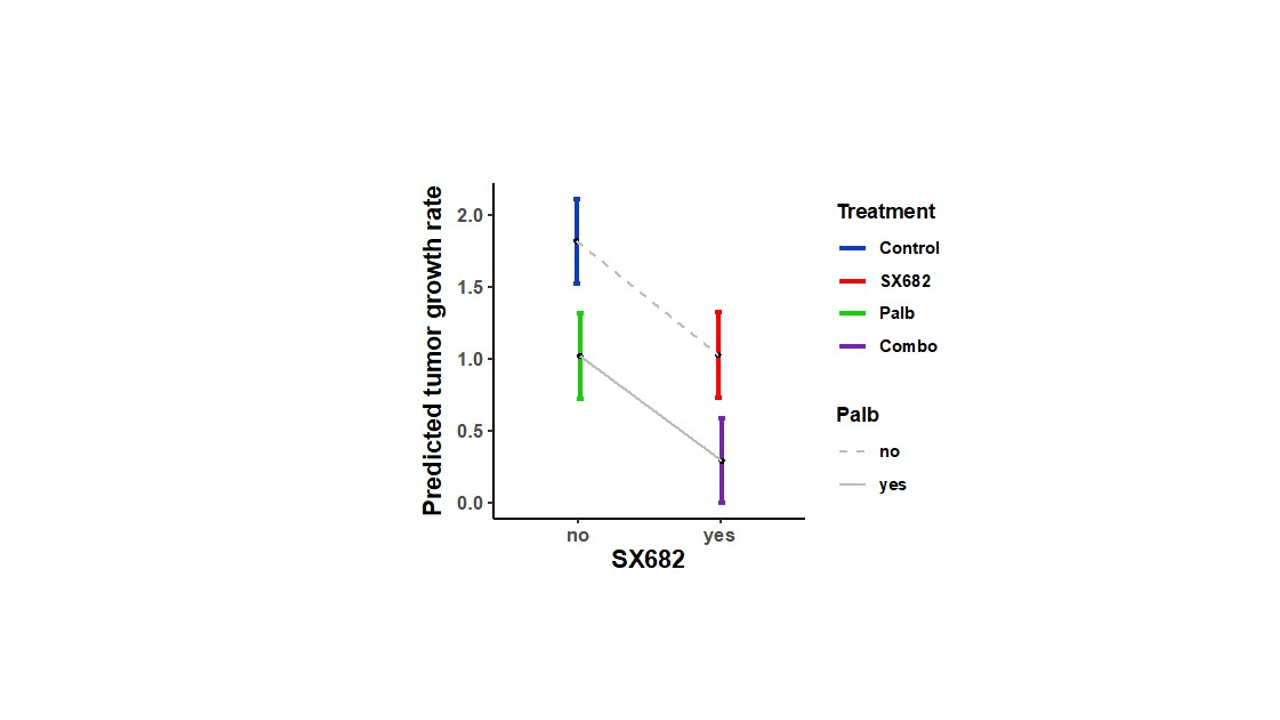

Supplement: Supplementary file 2 [file Image2.tif]

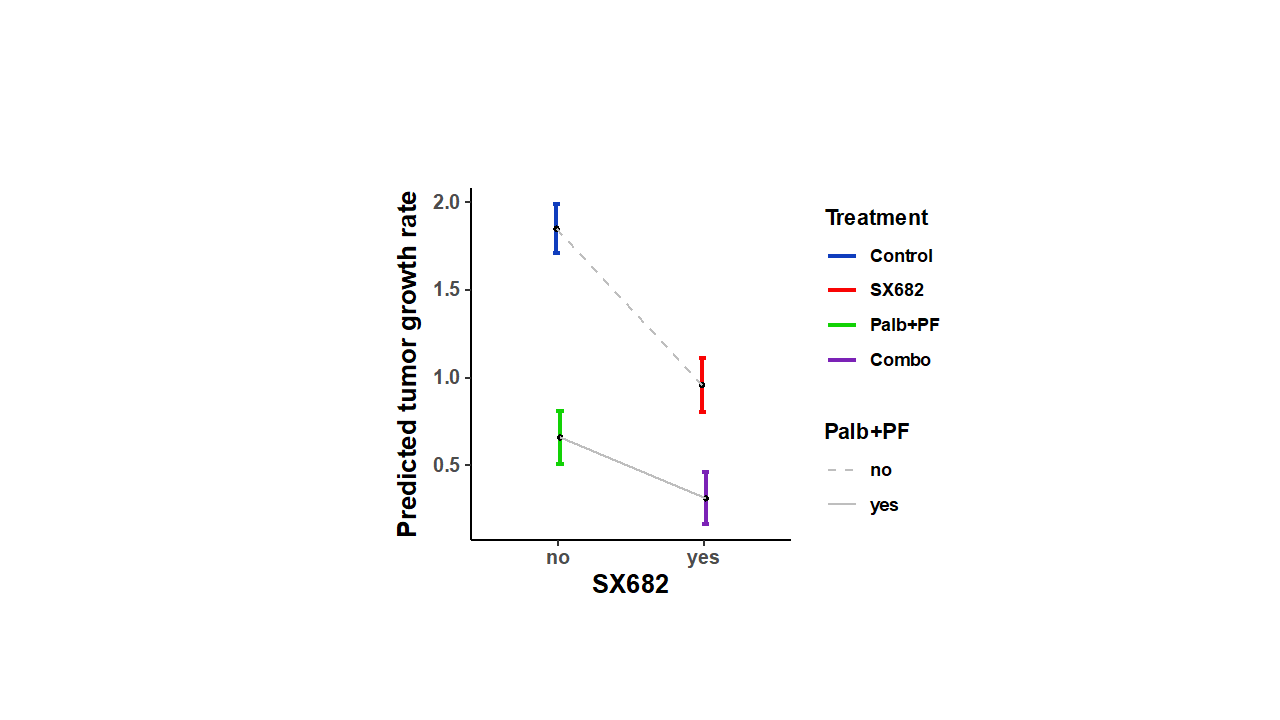

Supplement: Supplementary file 3 [file Image3.tif]
